# Reproductive outcomes in women with BRCA 1/2 germline mutations: A retrospective observational study and literature review

**DOI:** 10.1515/med-2024-9999

**Published:** 2024-08-20

**Authors:** Miriam Dellino, Antonio D’Amato, Gaia Battista, Gennaro Cormio, Antonella Vimercati, Vera Loizzi, Antonio Simone Laganà, Gianluca Raffaello Damiani, Alessandro Favilli, Sandro Gerli, Daniele La Forgia, Antonella Daniele, Vittorio Agrifoglio, Ettore Cicinelli, Amerigo Vitagliano, Andrea Etrusco

**Affiliations:** Department of Biomedical Sciences and Human Oncology, Obstetrics and Gynecology Unit, University of Bari “Aldo Moro”, 70124 Bari, Italy; Gynecologic Oncology Unit, IRCCS Istituto Tumori “Giovanni Paolo II”, 70124 Bari, Italy; Unit of Obstetrics and Gynecology, “Paolo Giaccone” Hospital, Department of Health Promotion, Mother and Child Care, Internal Medicine and Medical Specialties (PROMISE), University of Palermo, 90127 Palermo, Italy; Department of Medicine and Surgery, University of Perugia, 06132 Perugia, Italy; Department of Gynecology and Obstetrics, Istituto Tumori Giovanni Paolo II, I.R.C.C.S, 70124 Bari, Italy

**Keywords:** BRCA mutations, reproductive outcomes, fertility preservation, breast cancer, ovarian cancer

## Abstract

**Objective:**

To evaluate the reproductive outcomes of patients bearing BRCA-1 and BRCA-2 mutations.

**Methods:**

In this retrospective observational cohort study, we assessed data from BRCA-1 and BRCA-2 carriers, analyzing demographics, oncological history, and reproductive outcomes. Statistical analysis compared BRCA-1 and BRCA-2 carriers. A thorough review of the literature was carried out.

**Results:**

Fifty-eight patients were included. BRCA-1 and BRCA-2 mutations were equally distributed. Eighty-nine pregnancies occurred in our series, hesitated in 73 live births and 19 miscarriages. Mean age at first and last pregnancy was 27.8 ± 4.8 and 31.6 ± 4.8 years old. Thirty-nine patients have had at least one live birth (67.2%). Mean number of live births was 1.9 ± 0.6. Live birth rate (LBR) was 81.1% and miscarriage rate was 32.8%. Spontaneous fertility was unaltered, as evidenced by high LBR. Subgroup analysis revealed no significant differences between BRCA-1 and BRCA-2 carriers.

**Conclusions:**

Our results shows that spontaneous reproductive outcomes in BRCA-mutated patients are reassuring. Despite evidence indicating a decrease in ovarian reserve among BRCA patients, this factor seems to not impact spontaneous fertility negatively. Further research is needed, and individuals with BRCA mutations should consider early family planning and fertility preservation in case of partner absence.

## Graphical abstract

Despite evidence indicating a decrease in ovarian reserve among BRCA patients, this factor seems to not impact their spontaneous fertility negatively.

## 1 Introduction

Approximately 5–10% of ovarian cancer (OC) and breast cancer (BC) are associated with inherited pathological variants in genes that increase susceptibility to cancer [1]. In a minority proportion, other types of female genital cancers may also be associated with BRCA mutations [2–4].

The most common pathogenetic variants involve the BRCA-1 and BRCA-2 genes and are estimated to affect between 1/300 and 1/800 individuals in the general population [5]. These genes have vital functions in the repair of double-stranded breaks (DSB) through the homologous recombination repair process [6]. The dysregulation of one of these genes is responsible for genomic instability, resulting in an increased risk of cancer [7].

Carriers of BRCA-1 and BRCA-2 mutations have a cumulative lifetime risk of developing BC and OC of up to 72–69% for BC and 44–17% for OC, respectively [8]. These types of oncogenic mutations in BRCA genes can occur as germline or somatic mutations. Germline mutations are responsible for BRCA-1 and BRCA-2-associated hereditary breast and ovarian cancer and follow autosomal dominant inheritance. Somatic mutations, on the other hand, characterize cases of “BRCAness” or “BRCA-like” and are not subject to vertical transmission [9,10].

Regarding OC, the vast majority of BRCA mutations are germline, while somatic mutations are observed in around 5–7% of cases [11]. On the other hand, approximately 1–5% of all individuals diagnosed with BC possess a germline mutation in either the BRCA-1 or BRCA-2 genes [12,13], while somatic mutations are found at about half the prevalence of germline mutations [14,15]. The type of mutation is not the only determining aspect when assessing cancer risk in offspring inheriting the BRCA-1 or BRCA-2 pathogenic variant. In fact, multiple factors come into play, including the penetrance of the pathogenic variant, as well as the gender and age of the individual carrying the variant [16].

According to the guidelines of the National Comprehensive Cancer Network, it is recommended that women who carry BRCA mutations consider undergoing risk-reducing salpingo-oophorectomy (RRSO) either between the ages of 35 and 40 or after completing childbearing [17]. Regarding individuals with BRCA-2 mutations, it might be reasonable to postpone RRSO until they reach the ages of 40–45, taking into account the later onset of OC in this group [17]. In a recent study involving 3,517 women who received BRCA testing, the average age at diagnosis of OC was 51.3 years for BRCA-1 and 58.3 years for BRCA-2 [18]. Evidence from multiple studies suggests that RRSO is linked to a reduction in the occurrence of OC and BC, together with a significant decrease in overall mortality rates [19,20]. Several studies have shown that carriers of the BRCA-1 mutation might experience impaired fertility and reproductive potential. *In vitro*, oocyte-specific knockdown of key genes involved in DSB repair (BRCA-1, ATM, MRE11, Rad51, but not BRCA-2) resulted in an increase in DNA breaks, eventually leading to lower primordial follicle counts and reduced survival, both in human and murine cells [21].

Alongside ovarian aging, the mutations could also be responsible for an increased rate of aneuploidy, since BRCA-1 and ATM are implicated in meiotic spindle assembly and cohesion between sister chromatids [22,23]. During oocyte cryopreservation cycles, BRCA-mutated patients had worse results in terms of retrieved oocytes compared to BRCA-negative ones [24]. Finally, Porcu et al. demonstrated that BRCA-1 patients exhibited significantly lower levels of anti-Müllerian hormone (AMH) compared to BC patients without BRCA mutation and control women, concluding that BRCA-1 patients could have a higher risk of developing premature ovarian insufficiency (POI), together with a lower ovarian reserve [25]. In the same publication, BRCA-2 patients did not show the same decrease [25].

Recently, thanks to technological advances and increased awareness among practitioners, patients are diagnosed with the mutation earlier than in the past. Considering this, at the time of diagnosis, healthy carriers of BRCA are usually young and have to face different decisions on issues related to contraception, fertility, and pregnancy. Indeed, individuals carrying BRCA-1 or BRCA-2 mutations face unique challenges when making decisions regarding family planning, including concerns about pregnancy outcomes and long-term health implications [26].

To date, little information exists about the relationship between BRCA mutations and spontaneous fertility. Thus, the objective of the study is to evaluate the spontaneous reproductive outcome in a cohort of women carrying BRCA1/2 germline mutations.

## 2 Methods

### 2.1 Study design

This is a retrospective observational cohort study. The authorization of the ethics committee was not necessary because the data have been collected in a prospective manner on an anonymous database. The study did not mean any modifications in clinical practice for patients included.

Furthermore, a comprehensive literature search was conducted on the following databases: PubMed/MEDLINE, SCOPUS, The Cochrane Library, Science Direct, and Web of Science. All the relevant studies published till September 2023 were screened using relevant search terms as appropriate. Additional articles that met the inclusion criteria were identified by searching through the reference lists of the included articles. Articles that were not written in English or did not focus specifically on the topic were excluded.

The search was conducted independently by two reviewers, and further supervised by a third, reliable member of the research group. Any discrepancies were resolved by discussion. Data were extracted and summarized using a narrative synthesis approach.

### 2.2 Inclusion criteria

We included all patients diagnosed with germline mutations of BRCA-1 or BRCA-2 who were followed at the gynecological and obstetric clinic of the University Hospital of Bari, Italy from 2000 to 2023 for whom information about their reproductive history was available.

All patients who had other concurrent genetic mutations, a history of oncological treatments unrelated to the mutations of interest in the study, were excluded.

### 2.3 Data collection

Between the years 2000 and 2023, data belonging to women of reproductive age diagnosed with BRCA mutations at the University Hospital of Bari were retrospectively gathered.

Women carrying the mutation undergo annual routine monitoring at the oncological gynecology outpatient of our unit. During the visits, reproductive aspects were also investigated, with data collection conducted anonymously during the appointments. Various aspects such as patient demographics, clinical presentation, oncology marker levels, surgical procedures (and eventual risk-reducing surgery performed), histological examination, survival rates, and reproductive outcomes were extracted from hospital records.

The cancer history of each patient has been carefully collected, as well as the obstetrical medical history: for each patient, either data regarding previous cancers – in particular OC and BC; eventual previous or current chemotherapy; age at mastectomy or at RRSO; and number of pregnancies, miscarriages, live births were gathered.

Data regarding age at first and at last pregnancy, eventual surgery before or after pregnancy, number of spontaneous births and cesarean sections, and eventual complications during or after pregnancy were also thoroughly examined.

### 2.4 Statistical analysis

Statistical analysis was conducted using SPSS Statistics (v.22). Continuous variables were reported as mean ± standard deviation, while categorical variables were presented as frequencies (absolute) and percentages (%).

We compared the baseline characteristics and reproductive outcomes of patients with BRCA-1 and BRCA-2 mutations using the Student’s *t*-test for normally distributed continuous variables, and contingency tables with the chi-square test or Fisher’s exact test for categorical variables as appropriate. A *p*-value less than 0.05 was considered statistically significant.


**Ethical approval:** All procedures performed in this research were conducted in accordance with the 2013 Helsinki Declaration. Patients were informed about the protection of their data under the Privacy Act, and their written authorization was obtained specifically for the utilization of personal information only for scientific purposes.
**Informed consent:** Each patient was provided with a descriptive study form. Informed consent was obtained from all individuals who agreed to be part of the study.

## 3 Results

### 3.1 Included patients

Applying our inclusion and exclusion criteria, 58 patients were included in the present study, 29 of them carrying the BRCA-1 mutation (50%) and 29 bearing BRCA-2 mutation (50%).

Mean age of the patients was 41.8 ± 7.9 years old as on January 2023. Mean age at menarche was 12.15 ± 1.4 years old. Thirty-seven patients underwent RRSO (63.8%), while 21 have not yet decided to undergo surgery (36.2%). Four out of 58 patients decided to undergo risk-reducing mastectomy (RRM) (6.9%). Mean age at RRSO was 42.2 ± 4.1 years, while mean age at RRM was 36.5 ± 5.0 years. Twenty-five out of 58 subsequently developed BC and underwent radical mastectomy (43.1%). Of these, 20 BC (80%) occurred before RRSO. Two patients (8%) underwent simultaneously radical mastectomy and RRSO after being diagnosed with BC (patient 1 at the age of 41, BRCA-2 mutated and patient 2 at the age of 45, BRCA-1 mutated). Finally, three patients (12%) underwent radical mastectomy for BC, while they did not undergo RRSO yet. No BC occurred after RRSO in our cohort. Among the 25 patients who underwent radical mastectomy for BC, 14 occurred among BRCA-1 patients (56%) and 11 (44%) in BRCA-2 patients. The difference is not statistically significant. Since these were all adjuvant bilateral RRSOs, the average time between BC diagnosis and the RRSO was 66.8 ± 83.6 months. On the other hand, only one patient developed OC, and thus underwent subsequent debulking surgery and adjuvant chemotherapy (1.7%).

At the last follow-up visit, all patients were confirmed to be alive. No patient has taken or is currently taking hormonal replacement therapy (HRT) after RRSO. Additional data regarding patients’ medical background and surgery can be consulted in Table 1.

**Table 1:** Patients’ characteristics and data regarding demographics, medical background, surgery they have undergone

Patient characteristics	Data
No. of patients	58
BRCA-1 carriers	50% (29/58)
BRCA-2 carriers	50% (29/58)
Mean age, years (range)	41.8 ± 7.9 (19–50)
− BRCA-1	43.4 ± 5.9
− BRCA-2	40.8 ± 9.3
Mean age at menarche, years (range)	12.15 ± 1.4 (9–16)
− BRCA-1	12.2 ± 1.6
− BRCA-2	12.16 ± 1.3
Patients with history of RRSO (percentage)	63.8% (37/58)
− BRCA-1	54.1% (20/37)
− BRCA-2	45.9% (17/37)
Mean age at RRSO, years (range)	42.2 ± 4.1 (31–50)
− BRCA-1	42.2 ± 4.5
− BRCA-2	42.2 ± 3.7
Mean time from RRSO, months from today (range)	43.1 ± 23.4 (3–84)
− BRCA-1	44.4 ± 3.2
− BRCA-2	45.9 ± 25.5
Patients not undergoing RRSO (percentage)	36.2% (21/58)
− BRCA-1	47.6% (10/21)
− BRCA-2	52.4% (11/21)
Patients undergoing RRM (percentage)	6.9% (4/58)
− BRCA-1	50% (2/4)
− BRCA-2	50% (2/4)
Mean age at RRM, years (range)	37.25 ± 4.9 (31–43)
− BRCA-1	33.5 ± 5.0
− BRCA-2	40.5 ± 3.5
Subsequent BC with radical mastectomy (percentage)	43.1% (25/58)
− BRCA-1	56% (14/25)
− BRCA-2	44% (11/25)
Mean time from mastectomy, months from today (range)	90.7 ± 71.2 (3–300)
− BRCA-1	99.8 ± 78.2
− BRCA-2	99.2 ± 77.7
Subsequent OC (percentage)	1.7% (1/58)
− BRCA-1	100% (1/1)
− BRCA-2	0
Adjuvant chemotherapy (percentage)	1.7% (1/58)
− BRCA-1	100% (1/1)
− BRCA-2	0
Survival rate (percentage)	100% (58/58)
HRT	0 (0/58)

RRSO = risk-reducing salpingo-oophorectomy; RRM = risk-reducing mastectomy; BC = breast cancer; OC = ovarian cancer; HRT = hormonal replacement therapy.

### 3.2 Reproductive outcomes

Eighty-nine pregnancies occurred in our series, hesitated in 73 live births (67 from singleton pregnancies and 6 from twin pregnancies) and 19 miscarriages. Mean age at first and last pregnancy was 27.8 ± 4.8 and 31.6 ± 4.8 years for BRCA-1 and BRCA-2, respectively.

Thirty-nine patients have had at least one live birth (67.2%). The mean number of live births was 1.9 ± 0.6. Live birth rate (LBR) was 81.1% and miscarriage rate was 32.8%. Among the deliveries 43 occurred naturally (58.9%) and 30 via cesarean sections (41.1%).

No pregnancy occurred after RRSO. All pregnancies were reported spontaneous, and no patient reported having used assisted reproductive techniques (ARTs) to conceive.

Eleven patients developed complications of pregnancy (15.7%). Among these, one patient developed gestational cholestasis (1.4%), two patients fetal macrosomia (2.9%), one patient preeclampsia (1.4%), one patient anemia in pregnancy (1.4%), one patient fetal growth restriction (FGR) (1.4%), one patient placenta previa (1.4%), and one patient gestational diabetes (1.4%).

Data regarding obstetric outcomes and complications of the patients are summarized in Table 2.

**Table 2:** Summary of obstetric outcomes and complications of pregnancy of the patients

Obstetric outcomes	Data
Overall pregnancies, no.	89
Spontaneous pregnancies	100% (89/89)
ARTs pregnancies	0% (0/89)
Singleton pregnancies	75.3% (67/89)
Twin pregnancies	3.4% (3/89)
Live births, no.	73
Miscarriages, no.	19
Primiparous	17.2% (10/58)
Multiparous	50% (29/58)
Nulliparous	31% (18/58)
Mean age at first pregnancy, years (range)	27.8 ± 4.8 (17–36)
Mean age at last pregnancy, years (range)	31.6 ± 4.8 (18–40)
Mean number of live births (range)	1.9 ± 0.6 (1–3)
LBR	81.1%
Miscarriage rate	32.8%
Natural deliveries	58.9% (43/73)
Cesarean sections	41.1% (30/73)
Twin pregnancy	4.1% (3/73)

**Obstetric complications**	**Data (%)**
Obstetric complications	15.7% (11/70)
Fetal macrosomia	2.9% (2/70)
Gestational cholestasis	1.4% (1/70)
Preeclampsia	1.4% (1/70)
Anemia in pregnancy	1.4% (1/70)
FGR	1.4% (1/70)
Placenta previa	1.4% (1/70)
Gestational diabetes	1.4% (1/70)

ARTs = assisted reproductive techniques.

Obstetric complications are reported for the number of ongoing pregnancies, excluding the number of miscarriages.

### 3.3 Subgroup analysis

Subgroup analysis was conducted to investigate potential differences in various reproductive and demographic variables among patients with BRCA-1 and BRCA-2 mutations (Tables 3 and 4).

**Table 3:** Summary of BRCA-1 germline mutations and their functional implications in our cohort of patients

BRCA-1 mutation	Genomic mutation	Protein mutation	Functional impact	No. of patients
c.5329dupC; p.Gln1777ProfsTer74	c.5329dupC	p.Gln1777ProfsTer74	Frameshift, premature termination (Ter74)	10.3% (3/29)
c.1687C > T; p.Gln563Ter; rs80356898	c.1687C > T	p.Gln563Ter	Premature termination (Ter)	6.9% (2/29)
c.5266dupC; p.Gln1756Profs*74	c.5266dupC	p.Gln1756Profs*74	Frameshift, premature termination (Ter74)	20.7% (6/29)
NM_007294.4:c.5266dup; p.Gln1756ProfsTer74	NM_007294.4:c.5266dup	p.Gln1756ProfsTer74	Frameshift, premature termination (Ter74)	3.4% (1/29)
c.5266dupC	c.5266dupC	—	Frameshift, premature termination (Ter)	10.3% (3/29)
CNV	CNV	—	CNV	3.4% (1/29)
VUS of intron 5: c.301 + 7G > A	c.301 + 7G > A	—	VUS	3.4% (1/29)
c.65T > C; p.Leu22Ser	c.65T > C	p.Leu22Ser	Missense (Leu22Ser)	10.3% (3/29)
c.4484G > T; p.Arg1495Met	c.4484G > T	p.Arg1495Met	Missense (Arg1495Met)	3.4% (1/29)
c.190T > C; p.Cys64Arg	c.190T > C	p.Cys64Arg	Missense (Cys64Arg)	3.4% (1/29)
c.798_799delTT; p.Ser267Lysfs (rs80357724)	c.798_799delTT	p.Ser267Lysfs	Frameshift, premature termination (Terfs)	3.4% (1/29)
c.3228_3229delAG; p.Gly1077fs	c.3228_3229delAG	p.Gly1077fs	Frameshift, premature termination (Ter)	3.4% (1/29)
No data	—	—	—	17.2% (5/29)

VUS = variant of uncertain significance; CNV = copy number variation.

Mutations in the BRCA-1 and BRCA-2 genes were analyzed, with subsequent classification based on genomic and protein-level alterations.

**Table 4:** Summary of BRCA-2 germline mutations and their functional implications in our cohort of patients

BRCA-2 mutation	Genomic mutation	Protein mutation	Functional impact	No. of patients
c.1813dupA (p.Ile605Asnfs*11)	c.1813dupA	p.Ile605Asnfs*11	Frameshift, premature termination (Ter11)	3.4% (1/29)
c.1448_1451dupCAGT; p.Lys485Serfs*30	c.1448_1451dupCAGT	p.Lys485Serfs*30	Frameshift, premature termination (Ter30)	13.8% (4/29)
c.3029_3039delGAACAGCTTCA; p.Arg1010Lysfs*2	c.3029_3039delGAACAGCTTCA	p.Arg1010Lysfs*2	Frameshift, premature termination (Ter2)	6.9% (2/29)
c.6486_6489delACAA, p.Lys2162Asn*5	c.6486_6489delACAA	p.Lys2162Asn*5	Frameshift, premature termination (Ter5)	3.4% (1/29)
c.1796_1800del; p.(Ser599*). C.5200G > A; p.(Glu1734Lys)	c.1796_1800del; c.5200G > A	p.Ser599*; p.Glu1734Lys	Frameshift, premature termination (Ter), Missense (Glu1734Lys)	6.9% (2/29)
c.5796_5797delTA; p.His1932Glnfs*12	c.5796_5797delTA	p.His1932Glnfs*12	Frameshift, premature termination (Ter12)	10.3% (3/29)
c.859G > A (V211I)	c.859G > A	p.Val211Ile	Missense (Val211Ile)	3.4% (1/29)
c.2049_2050delTC; p.Gln684Glyfs*3	c.2049_2050delTC	p.Gln684Glyfs*3	Frameshift, premature termination (Ter3)	3.4% (1/29)
c.1976_1800delCT; p.Ser599fs1	c.1976_1800delCT	p.Ser599fs1	Frameshift, premature termination (Terfs*1)	13.8% (4/29)
c.2151T > A; p.Cys717Ter (rs876660512)	c.2151T > A	p.Cys717Ter	Premature termination (Ter)	3.4% (1/29)
c.2151T > C; p.Cys717X of exon 11	c.2151T > C	—	Premature termination (Ter)	3.4% (1/29)
c.9097dupA; p.(Thr3033Asnfs*11)	c.9097dupA	p.Thr3033Asnfs*11	Frameshift, premature termination (Ter11)	3.4% (1/29)
c.2045_2046delTC; p.Gln684GlyfsTer3	c.2045_2046delTC	p.Gln684GlyfsTer3	Frameshift, premature termination (Ter3)	3.4% (1/29)
p.Ser599*	—	p.Ser599*	Premature termination (Ter)	3.4% (1/29)
VUS c.91T > G; p.Trp31Gly in heterozygosis	c.91T > G	p.Trp31Gly	VUS	3.4% (1/29)
c.6131G > C; p.Gly2044Ala	c.6131G > C	p.Gly2044Ala	Missense (Gly2044Ala)	3.4% (1/29)
c.6468_6469delTC; p.Gln2157IlefsTer18	c.6468_6469delTC	p.Gln2157IlefsTer18	Frameshift, premature termination (Ter18)	3.4% (1/29)
No data	—	—	—	6.9% (2/29)

VUS = variant of uncertain significance.

Specifically, we examined age, age at menarche, age at mastectomy, pregnancies, full-term births, preterm births, miscarriages, live births, age at first pregnancy, age at last pregnancy, number of spontaneous births, and number of cesarean sections. For each of these variables, we performed independent sample *t*-tests to compare the means between the two subgroups, considering both cases with presumed equal variances and those with non-presumed equal variances.

The results, as shown in Table 5, indicate that there were no statistically significant differences in these variables between the two subgroups.

**Table 5:** Results of independent samples *t*-tests for reproductive and demographic parameters among BRCA-1 and BRCA-2 patients

Statistical test	No. of cases (*N*)	Assumption of equal variances	Significance (two-tailed)	Mean difference	Standard error of difference
Age	BRCA-1: *n* = 29	Assume equal variances	0.162	2.9138	2.0579
	BRCA-2: *n* = 29	Do not assume equal variances	0.167	2.9138	2.0737
Age at menarche	BRCA-1: *n* = 29	Assume equal variances	0.861	0.105	0.591
	BRCA-2: *n* = 29	Do not assume equal variances	0.871	0.105	0.634
Age at mastectomy	BRCA-1: *n* = 15	Assume equal variances	0.587	−0.857	1.560
	BRCA-2: *n* = 14	Do not assume equal variances	0.587	−0.857	1.558
Pregnancies	BRCA-1: *n* = 29	Assume equal variances	0.482	−0.258	0.365
	BRCA-2: *n* = 27	Do not assume equal variances	0.490	−0.258	0.371
Full-term births	BRCA-1: *n* = 29	Assume equal variances	0.842	0.054	0.269
	BRCA-2: *n* = 27	Do not assume equal variances	0.843	0.054	0.270
Preterm births	BRCA-1: *n* = 29	Assume equal variances	0.520	−0.040	0.061
	BRCA-2: *n* = 27	Do not assume equal variances	0.525	−0.040	0.062
Miscarriages	BRCA-1: *n* = 29	Assume equal variances	0.239	−0.203	0.171
	BRCA-2: *n* = 27	Do not assume equal variances	0.246	−0.203	0.173
Live births	BRCA-1: *n* = 29	Assume equal variances	0.754	0.086	0.272
	BRCA-2: *n* = 27	Do not assume equal variances	0.755	0.086	0.273
Age at first pregnancy	BRCA-1: *n* = 28	Assume equal variances	0.779	1.071	3.792
	BRCA-2: *n* = 26	Do not assume equal variances	0.779	1.071	3.793
Age at last pregnancy	BRCA-1: *n* = 28	Assume equal variances	0.932	0.365	4.271
	BRCA-2: *n* = 26	Do not assume equal variances	0.932	0.365	4.279
Number of spontaneous births	BRCA-1: *n* = 29	Assume equal variances	0.391	−0.234	0.270
	BRCA-2: *n* = 27	Do not assume equal variances	0.398	−0.234	0.274
Number of cesarean sections	BRCA-1: *n* = 28	Assume equal variances	0.460	0.164	0.220
	BRCA-2: *n* = 27	Do not assume equal variances	0.459	0.164	0.220

The significance level (two-tailed) indicates the *p*-value for each test. Mean differences and standard errors of differences are also provided. The tests assume equal variances in one set of data and do not assume equal variances in the other.

The lack of statistically significant differences suggests that the assumption of equal variances does not significantly impact the results for these reproductive and demographic variables.

## 4 Discussion

Healthy individuals and cancer patients who are carriers of germline pathogenic variants in BRCA-1/2 genes face multiple reproductive challenges that require appropriate counseling and expertise. As previously mentioned, most of the available preclinical evidence suggests that BRCA mutations could directly accelerate ovarian aging, reducing ovarian reserve both quantitatively and qualitatively [27,28].

On the other hand, our study, although on a small sample, shows that the spontaneous reproductive outcomes in BRCA-mutated patients are reassuring. The average number of children in this cohort 1.9 ± 0.6, and at least one live birth occurred in 67.2% of the patients. Only one case of infertility was certified, and the patient underwent IVF without success. The rest of the women did not attempt to have children. These data are consistent with those in the general population and with other studies in the same population [29,30]. Furthermore, the mean age at the first pregnancy in our cohort was 27.8 ± 4.8, which is lower than the Italian national average calculated in 2021, as well as the average number of children per woman being higher than 1.25, which is the latest Italian average [31]. It might be concluded, then, that while there may be a lower ovarian reserve in BRCA carriers compared to the general population according to the current evidence, this factor does not appear to negatively affect spontaneous fertility. Probably, the reduction in ovarian reserve is not reflected in fertility impairment if patients bearing BRCA mutations conceive at a younger age.

Provocatively, some have even discussed the possibility of heightened fertility in these women, countering the mutation’s impact, as if aiming to maximize reproductive potential within a narrower reproductive window [32]. Moreover, regarding spontaneous conception, our limited dataset would show that women carrying the mutation exhibit a higher average number of offspring compared to non-affected ones. This result appears in line with studies with greater statistical power [32].

Another major concern among carriers of pathogenic BRCA-1/2 variants is the potential higher risk of premature ovarian failure (POI) [33]. In our series we have not observed any case of POI.

How do we counsel a patient with a known BRCA germline mutation about fertility then? While in the past we have been addressing these women at the time of cancer diagnosis, their offspring will already be genetically mapped. This represents a significant aspect of the topic. While the current goal is primarily on mapping these patients, soon the role of the practitioner will shift toward counseling individuals who are already aware of their mutation from childhood, or even before birth.

At present, more emphasis should be given on advising women, from a reproductive perspective, to plan their families early in life. In cases of partner absence, cryopreserving their oocytes at a young age may be necessary, potentially requiring multiple controlled ovarian stimulation (COS) cycles, as their ovarian reserve diminishes more rapidly than in other women [25]. A timely execution and a greater consideration of prophylactic procedures should be considered mandatory in these patients. Should this be the way forward, every strategy aimed at preserving the best quality oocytes possible must be implemented [34,35].

If early-stage ovarian malignancy is detected, patients can be referred for fertility-sparing treatments. However, as with other fertility-sparing treatments for gynecologic malignancies [36–38], which, thanks to new technologies applicable to surgery [39,40], are increasingly effective, these patients should also be referred for radical surgery once the desire for offspring is satisfied. To date, it would also seem that fertility or endocrine function preservation techniques such as preservation of ovarian tissue and its subsequent re-implantation would not be recommended for patients with BRCA mutations [41].

As discussed earlier, BRCA1 and 2 are known to be involved in the DNA repair mechanism, through ATM-mediated regulation of DSB repair [6,7]. DBS DNA repair mechanisms play an important role in ovarian aging. A decrease in their efficiency causes not only an accelerated apoptotic loss of follicles with lethal mutations, but also an increase in meiotic errors and a reduced quality of oocytes, with an increase in the number of aneuploidies [22]. There is preclinical evidence that transgenic mice with defective BRCA genes have a reduced ovarian response to stimulation and decreased reproductive potential [21].

AMH levels are considered a quantitative marker of ovarian reserve, although not predictive of the possibility of spontaneous pregnancy [42]. In some studies, AMH levels were significantly lower in women carrying pathogenic variants in BRCA-1 or BRCA-2 or both genes [43], while other studies have reported no significant differences with controls [44,45].

Clinical studies describing a decrease in oocyte quality in human carriers of pathogenic BRCA variants (i.e., an increase in aneuploidies) are still lacking, while age at natural menopause among BRCA carriers is difficult to ascertain, due to various types of selection bias, different control groups, and small study population [46].

An additional problem related to the shortened window of reproductive opportunities is the recommendation of RRSO at a young age [5]. In our series, no patient has gone through menopause yet. 37/58 underwent RRSO (63.8%), while 21/58 did not undergo risk-reducing surgery yet (36.2%). In the first group, mean age at RRSO was 42.1 years for BRCA-1 and 42.3 for BRCA-2. Mean time from RRSO was 43.1 months from today. Although salpingectomy with delayed oophorectomy has been suggested as an option to preserve ovarian function, this strategy is not the gold standard and should not be recommended [47].

There are limited data on fertility outcomes in patients with BRCA-mutated BC. Oktay et al. first described in 2010 reduced ovarian response and lower oocyte count for fertility preservation in patients with BRCA-mutated cancer [48]. Since then, some other studies have found a worse quantitative response to COS in this cohort [48], while others reported no difference with non-mutated BC patients [49]. Similarly, some studies have reported lower levels of AMH at BC diagnosis in BRCA-mutated patients [21], while others did not report a significant difference [49]. Given these different problems, healthy carriers should be advised not to delay pregnancy beyond 35 years of age [50].

In addition, several studies have investigated the effect of parity on BC risk in healthy carriers of pathogenic BRCA variants [51]. About this topic, our study shows that most patients were diagnosed with BC after having at least one live birth (21/25 – 84%). A large prospective study showed that women with pathogenic BRCA-1 variants who had two, three, four, or more full-term pregnancies were at 21, 30, and 50% decreased risk of BC compared to women with a single full-term pregnancy [51]. In contrast, women with pathogenic BRCA-2 variants with multiple pregnancies had a significantly higher risk of developing BC [51]. However, this topic remains controversial due to the differences reported in carriers of pathogenic variants BRCA-1 and BRCA-2 and the different effects of pregnancy on the risk of BC according to age even in the general population not carrying BRCA mutations. In addition, it must also be considered that in the general population, healthy women have a transient increased risk of BC after pregnancy and the increased risk is greater for women with a family history of BC and for women with a pregnancy in old age [52]. This increased risk was attributed to the growth-promoting effect of the endocrinological environment of pregnancy on existing pre-malignant or malignant BC lesions that occur more frequently in later life [52]. In addition, differences between BRCA-1 and BRCA-2 carriers have been found in the literature in relation to the effect of breastfeeding on BC risk. In carriers of pathogenic variants BRCA-1, breastfeeding for at least 1 year reduces the risk of BC (OR = 0.68; 95% CI from 0.52 to 0.91; *P* = 0.008), while no effect has been described for healthy BRCA2 carriers [53].

Therefore, the timing of bilateral RRM in healthy BRCA carriers remains a matter of debate. On the one hand, breast removal before pregnancy constantly reduces the risk of subsequent BC and the need for careful monitoring during pregnancy and lactation.

On the other hand, many women highly appreciate the benefits of breastfeeding their babies and are reluctant to undergo surgery before pregnancy. For the considerations mentioned above, it is clear that the counseling of young carriers of BRCA pathogenic variants seeking pregnancy is complex and should consider risk estimates based on family history, age, and breast density, but also personal values on reproductive choices, which remain extremely sensitive and personal [26].

The woman chooses to keep the breast and postpone bilateral RRM, careful monitoring with breast ultrasound during pregnancy and with breast ultrasound and mammography during lactation should be planned. In addition, none of our patients had pregnancy after BC. In this group of patients, current data show that subsequent pregnancy does not increase BC-related events [54]. In BC patients requiring 5–10 years of adjuvant endocrine therapy (ET) including BRCA carriers, an international clinical trial, which recently completed target accumulation, is evaluating the safety and feasibility of temporary interruption of ET after at least 18 months, in order to enable pregnancy [55].

In addition, a large international study has recently shown that, regardless of receptor status and particularly for carriers of pathogenic BRCA-1 variants, pregnancy after BC appears to be safe without negative consequences on maternal prognosis or fetal outcomes [56]. Although this study included a significant number of patients, it had some limitations including short-term follow-up (∼4 years follow-up from pregnancy) and limited power to detect differences particularly in BRCA-2 carriers [56]. Moreover, the retrospective nature of this and other studies represents an important limitation that does not allow definitive and strong conclusions to be drawn. Another data to be considered is represented by the high BC rate (43.1%) in our cohort. This parameter is likely attributed to the fact that more than half of the patients received their BRCA diagnosis following a BC diagnosis.

The main limitations of our study were the small sample size and the retrospective design. The main strengths of this study include the long follow-up period, the rigorous sample selection, and its originality.

In conclusion, spontaneous fertility remained unaffected in our cohort of women with BRCA germline variants. These findings are promising and hold significant implications for counseling young patients with BRCA mutations. While there is considerable interest in this field, the absence of large prospective studies on these subjects underscores the necessity for further research endeavors. Moreover, misconceptions persist among healthcare professionals and patients, often resulting in fragmented and suboptimal care.

Since the reproductive window may be narrower compared to the general population, individuals carrying BRCA germline variants should be encouraged to complete their family planning at a younger age and undergo comprehensive breast follow-up examinations during pregnancy and breastfeeding if they choose to retain their breasts. Nevertheless, for patients with BRCA-mutated BC, subsequent pregnancies after receiving appropriate treatment and follow-up do not seem to increase the risk of BC recurrence and should not be discouraged.
